# Fabrication, Properties, and Biomedical Applications of Calcium-Containing Cellulose-Based Composites

**DOI:** 10.3389/fbioe.2022.937266

**Published:** 2022-06-20

**Authors:** Ru-Jie Shi, Jia-Qi Lang, Tian Wang, Nong Zhou, Ming-Guo Ma

**Affiliations:** ^1^ Chongqing Engineering Laboratory of Green Planting and Deep Processing of Famous-region Drug in the Three Gorges Reservoir Region, College of Biology and Food Engineering, Chongqing Three Gorges University, Chongqing, China; ^2^ Research Center of Biomass Clean Utilization, Engineering Research Center of Forestry Biomass Materials and Bioenergy, Beijing Key Laboratory of Lignocellulosic Chemistry, College of Materials Science and Technology, Beijing Forestry University, Beijing, China

**Keywords:** cellulose, composites, hydroxyapatite, calcium carbonate, biomedical application

## Abstract

Calcium-containing cellulose-based composites possess the advantages of high mechanical strength, excellent osteoconductivity, biocompatibility, biodegradation, and bioactivity, which represent a promising application system in the biomedical field. Calcium-containing cellulose-based composites have become the hotspot of study of various biomedical fields. In this mini-review article, the synthesis of calcium-containing cellulose-based composites is summarized via a variety of methods such as the biomimetic mineralization method, microwave method, co-precipitation method, hydrothermal method, freeze-drying method, mechanochemical reaction method, and ultrasound method. The development on the fabrication, properties, and applications of calcium-containing cellulose-based composites is highlighted. The as-existed problems and future developments of cellulose-based composites are provided. It is expected that calcium-containing cellulose-based composites are the ideal candidate for biomedical application.

## 1 Introduction

Cellulose received considerable attention due to its properties of mechanical strength, biocompatibility, and biodegradation and its wide applications in clothing, paper, biofuel, and biomedical fields (Eichhorn e*t al.,* 2010; Moon et al., 2011; Huang et al., 2020,^2022^; Wang et al., 2021; Kang et al., 2022). There are more than 30,000 studies that used “***cellulose***” as the “***title***” on the Web of Science over the last 10 years, indicating that cellulose has become a hot research topic. Calcium-containing inorganic functional materials mainly included hydroxyapatite (Ca_10_(PO_4_)_6_(OH)_2_, HA), CaCO_3_, calcium silicate, and CaSO_4_ (Tran and Webster, 2009). HA is used in the fields including drug delivery, toothpaste additive, and dental implants because of its biocompatibility, bioactivity, and biological properties (Suchanek and Yoshimura, 1998; Chu et al., 2002; Ma et al., 2006; Ma and Zhu, 2009; Ma, 2012). Moreover, carbonated hydroxyapatite (CHA), containing carbonate ions of 6∼8 mass%, shows high bioactivity in comparison to that of HA (Gibson and Bonfield, 2002; Landi et al., 2003; Morales-Nieto e*t al.,* 2013). CaCO_3_ is abundant in organisms (Politi et al., 2004). The calcium-containing cellulose-based composites combined the characteristics of cellulose and calcium-containing inorganic materials, producing new properties by synergistic effect **(** Hokkanen et al., 2016 **)**. Therefore, it is expected that calcium-containing cellulose-based composites meet the requirements of applications. As early as 2010, the progress in the fabrication of calcium-based inorganic biodegradable nanomaterials was reviewed by Ma and Zhu (2010). Qi *et al.* (2018) reviewed the synthesis and properties of calcium-based biomaterials for diagnosis, treatment, and theranostics. In the previous review study, we summarized the recent development of multifunctional cellulose and cellulose-based nanocomposites adsorbents (Shi et al., 2022).

In recent years, there are rapid demands for biomedical materials (Habraken et al., 2016; Hu et al., 2018; Fu et al., 2019c; Yi et al., 2020; Yuan et al., 2021). For example, the disabled and bone injury patients need a lot of bone repair materials, the patients with cardiovascular disease need artificial heart valves, and the patients with renal failure need kidney dialyzers. It was reported that the composites, consisting of inorganic materials such as HA, CaCO_3_, calcium silicate, SiO_2_, and polymer including collagen, chitosan, chitin, hyaluronic acid, cellulose, poly lactic acid (PLA), poly glutamic acid (PGA), and poly caprolactone (PCL), were the new generation biomedical materials in the 1990s, which had the features of bioactivity and biodegradability to meet the clinical needs (Kaur et al., 2015; Liu et al., 2020). Chan *et al.* (2002) reported the synthesis of polypropylene/calcium carbonate nanocomposites. In fact, it is believed that cellulose is an organic biomedical material, meanwhile, calcium-containing inorganic functional composites are inorganic biomedical materials. The calcium-containing cellulose-based composites are promising biomedical materials to meet the requirements of applications (Oprea and Voicu, 2020).

This current mini-review study gives an overview of the synthesis, properties, and applications of calcium-containing cellulose-based composites. In section two, various methods including the biomimetic method, the microwave method, the co-precipitation method, the hydrothermal method, the freeze-drying method, the mechanochemical reaction method, and the ultrasound method were summarized for the synthesis of calcium-containing cellulose-based composites. In section three, the properties of calcium-containing cellulose-based composites such as mechanical properties, degradation, bioactivity, biocompatibility, feasibility, viability, cytocompatibility, cell-guiding property, antibacterial properties, and ion-exchangeability were also reviewed. In addition, the applications of these composites were described in the tissue engineering scaffolds, histological, drug delivery, and wastewater treatment. Finally, the future developments of calcium-containing cellulose-based composites were suggested.

### 1.1 Synthesis of Calcium-Containing Cellulose-Based Composites

#### 1.1.1 Biomimetic Mineralization Method

Biomineralization refers to the generation process of inorganic minerals by the biological macromolecules of the organism (Addadi, Raz, and Weiner, 2003). In comparison to general mineralization, the process of biomineralization involved the biological macromolecules, cells, and organic matrix (Gower, 2008). Based on the biomineralization mechanism, the biomimetic synthesis method is an important route for creating biomedical materials by imitating the synthetic process of natural reaction and structure (Dorozhkin, 2011). The biomimetic synthesis method was developed to fabricate biomedical materials (Tas, 2000). Addadi, Raz, and Weiner (2003) reviewed the amorphous calcium carbonate by the biomineralization method.

Fang *et al.* (2009) prepared bacterial cellulose (BC)/HA nanocomposite scaffolds *in vitro* biocompatibility by the biomimetic technique. As for the fabrication of cellulose/HA composites via the biomimetic method, Wan and coworkers had done system work. In 2006, they developed the biomimetic precipitation of CHA with low crystallite size and crystallinity on BC from simulated body fluid (SBF) (Hong et al., 2006). Then, the biomimetic method was reported to synthesize CHA/BC composites by soaking phosphorylated and CaCl_2_-treated BC fibers in the SBF (Wan et al., 2006). After that, they applied the biomimetic method to fabricate CHA/BC nanocomposites with a three-dimensional (3D) network and crystallinities below 1% (Wan et al., 2007). It found the formation of HA on BC in the existence of phosphorylation. Furthermore, HA/BC nanocomposites were also carried out via the biomimetic route (Zhang et al., 2009). It carried out the growth of calcium phosphate via phosphorylation reaction.

Generally, HA has a ratio of 1.67 for Ca/P. However, calcium-deficient HA is always obtained with a ratio of Ca/P below 1.67 in nature. Hutchens *et al.* (2006) first synthesized calcium-deficient HA in the BC hydrogel. BC was used as a template for the biomimetic fabrication of apatite. Shi *et al.* (2009) synthesized calcium-deficient HA/BC nanocomposites with improved mineralization efficiency by combining the alkaline treatment and biomimetic mineralization process. Zimmermann and coworkers (Zimmermann et al., 2011) designed calcium-deficient HA/BC nanocomposites using the biomimetic approach in dynamic SBF for bone healing applications. Hammonds *et al.* (2012) prepared calcium-deficient HA/BC composites.

It reports that SBF is a very important media for the formation of HA during the process of biomimetic mineralization. The synthesis of cellulose fabrics with hydroxy carbonated apatite using the biomimetic method in SBF was reported by Hofmann *et al* (2006). Cromme et al. (2007). In Cromme’s study (2007), regenerated cellulose (RC) films were obtained with hydrochloric acid vapors, in which the calcium phosphate was formed in SBF. Yin *et al.* (2011) prepared HA/BC nanocomposites by the biomimetic mineralization method. It found that CHA nanorods were grown *in vitro* along with the network of BC via the dynamic SBF treatment. Rodriguez, Renneckar, and Gatenholm (2011) used the electrospinning method to produce cellulose acetate (CA) scaffolds. Li *et al.* (2012) synthesized electrospun cellulose nanofiber (CNF)/HA composites with micro-, meso-, and macro-pores in SBF. It achieved the growth of HA along the fibers in the composites. Petrauskaite *et al.* (2013) developed biomimetic mineralization using the cellulose porous matrix in the SBF solution. It achieved the improved cell adhesion and growth rate on the porous cellulose matrix. Garai and Sinha (2014) reported the biomimetic synthesis of 3D micro/macro CMC/HA nanocomposites. 3D nanocomposite structures were due to the ionic/polar or electrostatic interactions of HA impregnated CMC matrix. It obtained compressive strength of 1.74–12 MPa and a compressive modulus of 157–330 MPa.

**FIGURE 1:**
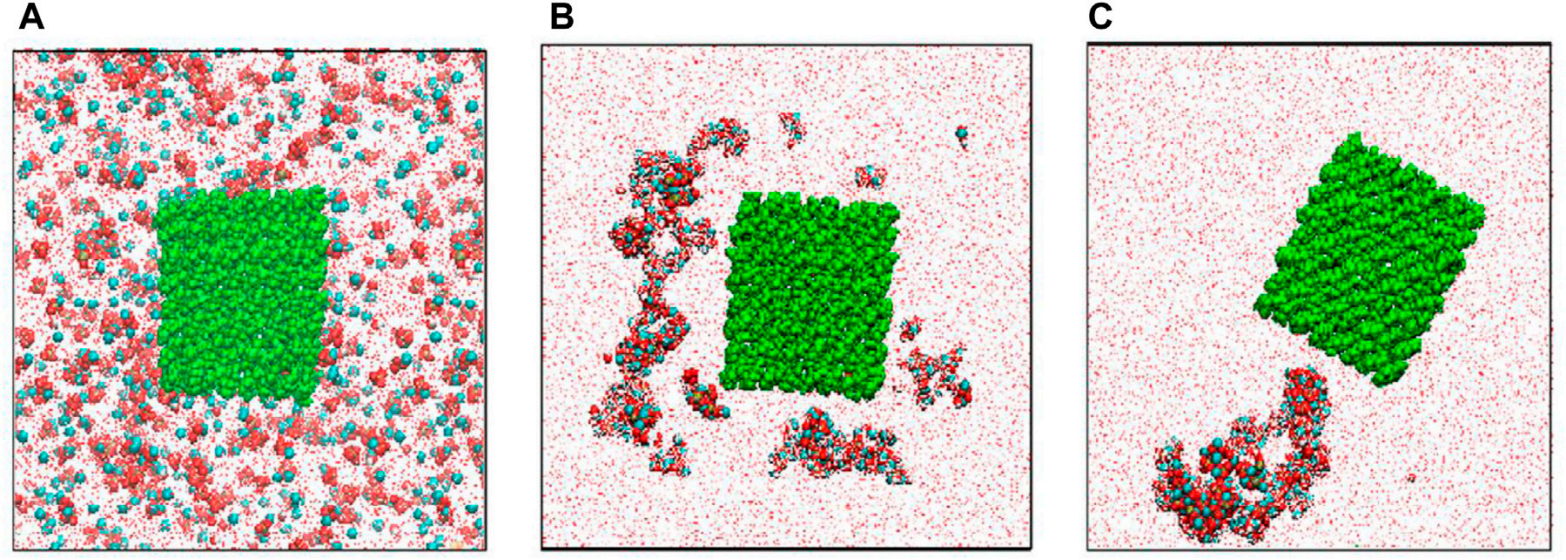
Distributions of ions in the modeling cell: **(A)** in the initial state, **(B)** in 12 ns, and **(C)** at the end (100 ns) of simulation. Red balls-O atoms, cyan balls-Ca^2+^ ions, yellow balls-P atoms, white balls-H atoms, green balls-cellulose atoms (Lukasheva and Tolmachev, 2016).

Lukasheva and Tolmachev (2016) presented biomimetic synthesis and molecular dynamics simulation of HA/BC nanocomposites. The CP crystals nucleate initially in solution, and then adsorbed on the surfaces of BC nanofibrils (Figure 1). Yang *et al.* (2016) applied the biomimetic process to prepare oxidized BC/HA/gelatin nanocomposites with the 3D network for a potential bone scaffold material. The nanocomposites have a tensile strength of 0.3 MPa and a complete degradation time of approximately 90 days in SBF. Kim *et al.* (2018) fabricated 3D pore-structure biomimetic cellulose/calcium-deficient HA composite scaffolds for bone tissue engineering. It found bone mineralization in the composite scaffold via cellular responses using preosteoblasts (MC3T3-E1). Liu *et al.* (2019) used BC hydrogel to synthesize biomimetic multilevel HA. It observed the weak coordination between the hydroxyl groups of BC molecule with Ca^2+^. Okuda *et al.* (2022) applied the biomimetic approach to preparing the CMC/HA composites with a stable interface. It achieved the flexural strength of 113 ± 2 MPa and the elastic modulus of 7.7 ± 0.3 GPa. It found an ionic interaction between Ca^2+^ and COO-.

**FIGURE 2:**
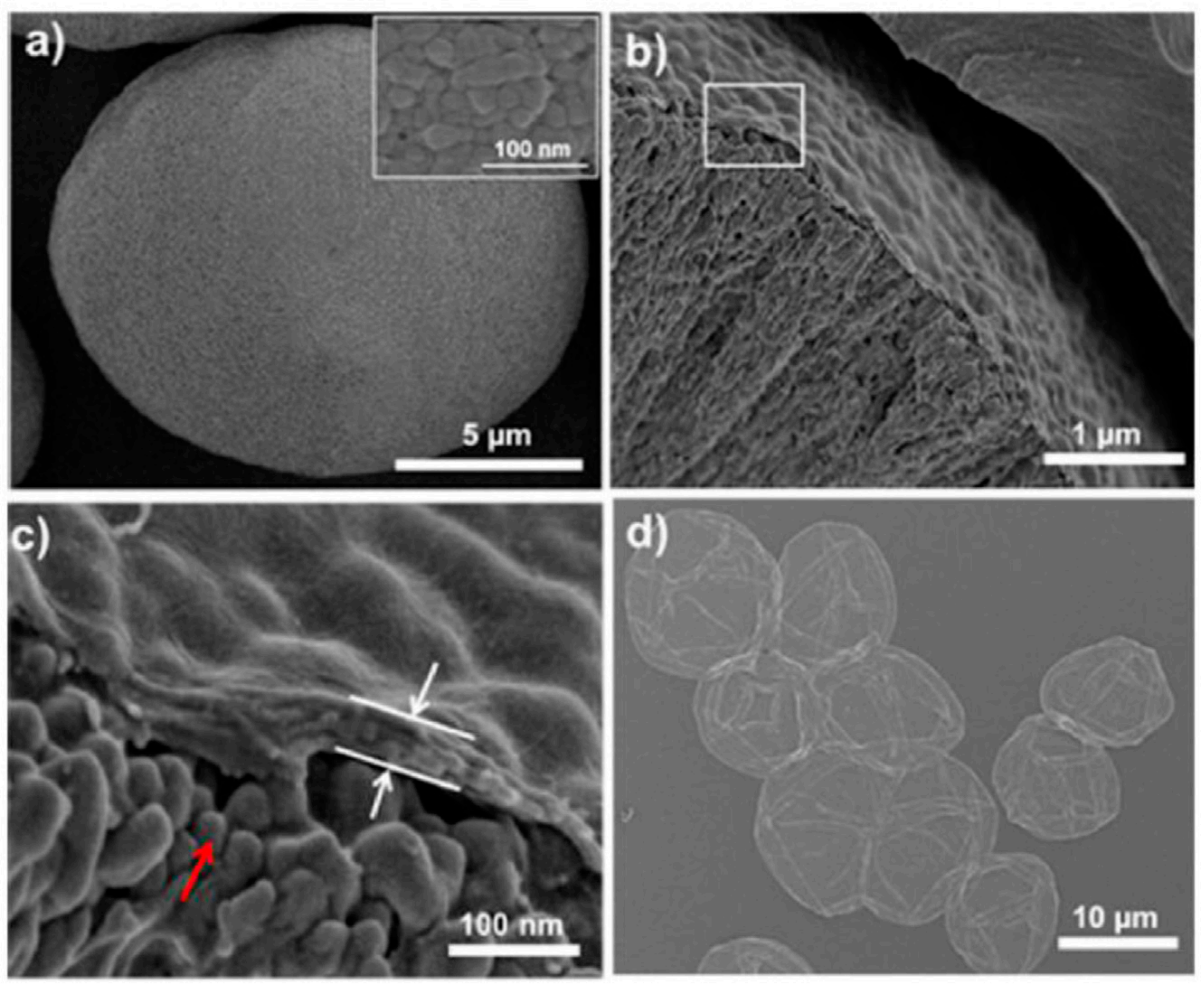
The SEM images of **(A)** spherical CaCO_3_ microparticles. The inset: details of the CaCO_3_ surface. **(B)** Cross-section of CaCO_3_ (AP/CNF)_5_AP/XyG microparticles, and **(C)** is a higher magnification of **(B)**. The red arrow points to the surface morphology of CaCO_3_ microparticles. The white arrows point to the AP/CNF multilayer. **(D)** The SEM image of (AP/CNF)_5_AP/XyG microcapsules after core removal (Paulraj et al., 2017).

Li and Wu (2009) reported the monodisperse rosette-like calcite mesocrystals in CMC by the biomimetic gas-diffusion method. Xiao *et al.* (2011) prepare calcium carbonate on RC fibers in ethanol-water mixed solvents by the mineralization method. It found that twin-sphere-based vaterite, zonary, and rod-like calcite were embedded in fibers. Liu *et al.* (2011) used electrospun CA fibers modified by poly acrylic acid (PAA) as scaffolds for the mineralization of CaCO_3_. It showed the calcite film coatings with needle-like shapes on the surfaces of CA fibers. The carboxylic groups of acidic PAA molecules interacted with the OH moieties of CA, then bent with Ca^2+^ ions on the surfaces of CA fibers. Rauch *et al.* (2012) synthesized calcite with minor fractions of aragonite on and in RC gel membranes by a diffusion-driven mineralization approach. The experimental result indicated that the calcium carbonates were assembled from building blocks. Liu *et al.* (2013) fabricated BC/lamellar CaCO_3_ hybrid induced by egg white *in situ* by the biomimetic mineralization method. The hybrid had a rough surface and an elaborate 3D structure with controllable porosity. Vyroubal *et al.* (2013) fabricated CaCO_3_ in BC by the biomimetic method. Paulraj *et al.* (2017) used CaCO_3_ as a template to fabricate microcapsules with controllable permeability properties by the layer-by-layer method in plant polysaccharides of pectin, cellulose nanofibers, and xyloglucan. It obtained the spherical CaCO_3_ with 16 ± 4 μm (Figure 2A), the CaCO_3_ (AP/CNF)_5_AP/XyG microparticles with a thickness of ∼60 nm (Figures 2B, C), and hollow microcapsule structures after complete core removal (Figure 2D).

The biomimetic mineralization method was widely used to synthesize biomaterials, induce bioactive materials, and investigate the synthetic mechanism. However, it needs long reaction times, complex reaction procedures, and precise control conditions. Recently, Qi *et al.* (2019) used the biomolecule-assisted green method for the synthesis of nanostructured calcium phosphates and investigated their biomedical applications.

#### 1.1.2 Microwave-Assisted Method

The microwave-assisted method is a greener technology due to its characteristics of reduced energy consumption, short reaction time, and high yield (Zhu and Chen, 2014). Over the past years, the microwave-assisted method was used to fabricate metals, metal oxides, and metal sulfides (Ma et al., 2014; Meng et al., 2016). In the several review articles, one can find the applications of the microwave-assisted method (Tsuji et al., 2005; Polshettiwar et al., 2009; Baghbanzadeh et al., 2011). For example, Tsuji *et al.* (2005) reviewed the development of the microwave-assisted synthesis of metallic nanostructures in solution. Polshettiwar *et al.* (2009) described the applications of the rapid and sustainable microwave-assisted route to synthesize organics and nanomaterials.

In the earlier studies, the cellulose/HA nanocomposites were fabricated by the microwave-assisted method (Ma et al., 2010). It found the homogeneous dispersion of HA nanoparticles with a narrow size distribution in the cellulose matrix. The cellulose/CHA nanocomposites were also obtained via the microwave-assisted method in the NaOH/urea solution (Jia et al., 2010b). The cellulose/CHA nanocomposites with a rough surface and aggregated CHA nanorods were also carried out in ionic liquid (IL) by the microwave-assisted method (Ma et al., 2011). It noted that F-substituted HA could enhance the acid resistance and stability of HA. The cellulose/F-substituted HA nanocomposites were also obtained in ILs via the microwave-assisted method (Jia et al., 2012a). It found the increased number of F-substituted HA with increasing heating time. The lignocellulose/HA nanocomposites were also carried out via the microwave-assisted rapid synthesis method (Fu et al., 2015). Both the morphologies and sizes of HA in the nanocomposites were adjusted via heating time. Fu *et al.* (2016) developed the microwave-assisted hydrothermal method for the synthesis of cellulose/HA nanocomposites using sodium dihydrogen phosphate dihydrate or adenosine 5-triphosphate disodium salt, creatine phosphate disodium salt tetrahydrate, or D-fructose 1,6-bisphosphate trisodium salt octahydrate. All the phases, sizes, and morphologies of the nanocomposites were affected by phosphate sources. It obtained various HA morphologies of nanorods, pseudo-cubic, pseudo-spherical, and nano-spherical particles.

The preparation of calcium sulfate nanowires was reported by thermal transformation of calcium dodecyl sulfate in the ethylene glycol and *N,N*-dimethylformamide mixed solvents (Li et al., 2008). The synthesis of cellulose/calcite composites was group explored using alkali extraction cellulose and MCC using the microwave-assisted method (Ma et al., 2012a). It achieved composites with better crystallinity using MCC than that of alkali extraction cellulose. Moreover, it found cellulose fibers and CaCO_3_ particles using alkali extraction cellulose, and irregular cellulose and CaCO_3_ microspheres using MCC. The cellulose/CaCO_3_ nanocomposites were formed in the alkali extraction of cellulose by the microwave-assisted IL method (Ma et al., 2013). IL acted as the solvent for absorbing microwave, dissolving cellulose, and synthesizing cellulose/CaCO_3_ nanocomposites, and it was found that the change morphologies of CaCO_3_ from polyhedral to cube to particle occurred with increasing cellulose concentration. Cytotoxicity experiments demonstrated the cellulose/CaCO_3_ nanocomposites with good biocompatibility. Cheng *et al.* (2016) used the microwave method to fabricate cellulose/CaCO_3_ composites in an IL/ethylene glycol mixed solution within 10 min. It was found that ILs favored the synthesis of composites. The microwave IL method was reported for the fabrication of cellulose/calcium silicate nanocomposites in ethylene glycol (Jia et al., 2011a). ILs had an effect on the composite of cellulose and calcium silicate. After that, the cellulose/calcium silicate nanocomposites were obtained by the microwave method in ILs and recycled ILs (Jia et al., 2011b). Both the size and microstructure of cellulose/calcium silicate nanocomposites were influenced by starting ILs and recycled ILs.

In general, the microwave method is green, rapid, and environmentally friendly for the synthesis of cellulose-based nanocomposites. In particular, considerable study should be carried out on the fabrication, structure, and property of cellulose/HA nanocomposites by the microwave-assisted method. This rapid microwave-assisted method is completely different from the aforementioned biomimetic synthesis method, but it could significantly shorten the reaction time and improve the reaction selectively and the yield, and be suitable for the large-scale synthesis in modern industrial production.

### 1.2 Co-Precipitation Method

As a traditional synthesis method, the co-precipitation method is an important strategy to obtain homogeneous composites with small size and narrow size distribution (Doerner and Hoskins, 1925; Park et al., 2003). The co-precipitation method is similar to the precipitation method, which is cumbersome and time-consuming. As early as 1925, the co-precipitation method was applied for the preparation of radium and barium sulfates by Doerner and Hoskins (1925).

Zakharov *et al.* (2005) obtained HA/CMC composites with a pore structure via the co-precipitation method for biomedical applications. Grande *et al.* (2009) synthesized the CHA/BC nanocomposite via a wet chemical precipitation method. In Kumar’s study (Kumar et al., 2010), the co-precipitation method was developed for the preparation of biomimetic CMC/HA nanocomposites. Nunez *et al.* (2020) used *in situ* wet chemical precipitation technique to synthesize BC/HA nanocomposite adsorbent. It carried out a removal capacity of 192 mg g^−1^ in batch experiments and 188 mg g^−1^ in packed-bed column systems for Pb(II). Tabaght *et al.* (2021) developed a dissolving and precipitation technique for the synthesis of biocompatible HA/cellulose composite for bone substitute. Sivasankari *et al.* (2021) reported a chemical precipitation technique to prepare HA incorporated CA/polyetherimide membrane with biocompatibility for adsorption and biomedical applications. The co-precipitation method is an important route for the preparation of HA/cellulose nanocomposites. It is known that the synthesis of HA is a double decomposition reaction with rapid nucleation and growth rate. So it is not easy to obtain homogeneous cellulose/HA composites by the co-precipitation method.

Ciobanu *et al.* (2010) reported the cellulose fibers with CaCO_3_ by the *in situ* precipitation method. CaCO_3_ was precipitated into the lumen and wall pores of fibers by the *in situ* precipitation method. The cellulose/calcium silicate nanocomposites were carried out by the precipitation method (Li et al., 2010). Stroescu *et al.* (2012) deposed CaCO_3_ on BC membranes using sodium dodecyl sulfate (SDS) and cetyl trimethylammonium bromide (CTAB) by a precipitation reaction. It obtained the calcium carbonate with rhombohedral and flower-like by adjusting the surfactant type and concentration. Zhu *et al.* (2020) prepared RC/calcium carbonate biocomposite films with flexibility, optical properties, mechanical strength, and thermal stability by *in situ* precipitation. It found a tensile strength of 84.7 ± 1.5 MPa for biocomposite.

### 1.3 Hydrothermal Method

The hydrothermal method refers to the synthesis of functional materials with water as the solvent in the vessel sealing at high temperature and high-pressure conditions. In the mid-19th century, geologists simulated the mineralization in nature and found the hydrothermal method. After 1900, the theory of hydrothermal synthesis was constructed. Then, the hydrothermal method was developed to synthesize the functional materials (Byrappa and Adschiri, 2007). It reported the hydrothermal synthesis of 0D, 1D, and 2D materials and composites (Feng and Xu, 2001).

The hydrothermal method of lignocelluloses had an effect on cellulose, hemicellulose, and lignin (Garrote et al., 1999). Jiang and Zhang (2009) prepared HA nanorods with well-controlled particle size and porosity through the hydrothermal method using phosphate ester as the structure-directing agent and sodium salt CMC as the template. The hydrothermal method was applied to prepare cellulose/CHA nanocomposites with CHA nanostructures dispersed in the cellulose matrix in a NaOH-urea aqueous solution (Jia et al., 2010a). In comparison with the biomineralization method, the hydrothermal method needs high temperature and high-pressure conditions, which restrain the wide application in the synthesis of biomedical composites. Moreover, Palaveniene *et al.* (2019) reported the hydrothermal synthesis of an osteoconductive 3D porous RC/HA composite scaffold with a porosity of 85% for bone tissue regeneration. The MG-63 cells proliferated well on scaffolds via *in vitro* cell culture. Pieper *et al.* (2020) prepared cellulose/HA biofilm with good thermal stability using a microwave-assisted hydrothermal synthesis at 140°C for 5 min.

The hydrothermal method was developed to obtain cellulose/CaCO_3_ bio-nanocomposites with good biocompatibility in the NaOH/urea solution (Jia et al., 2012b). The urea was also used as the CO_3_^2−^ source for the preparation of CaCO_3_. Furthermore, the fabrication of wood powder/CaCO_3_ composites was investigated by the hydrothermal method (Ma et al., 2012b). This work utilized all the main components of lignocelluloses, compared with cellulose-based composites.

#### 1.3.1 Other Synthesis Methods

The freeze-drying method might be more suitable for the synthesis of biomedical composites, which spray the solution to the organic liquid, then freeze instantaneous, sublimate, dehydrate, and decomposed to produce the comparatively loose products. The freeze-drying method was applied to synthesize cellulose/HA composites by Jiang group (Jiang L. et al., 2008). They incorporated CMC into HA/chitosan to obtain HA/chitosan/CMC composite as 3D scaffold by the freeze-drying method. Then, they applied the freeze-drying method for the preparation of the HA/chitosan/CMC porous composite scaffolds with different weight ratios (Jiang L.-Y. et al., 2008). After that, HA/chitosan and CMC composite scaffolds with good cell biocompatibility and tissue biocompatibility were also carried out by the freeze-drying method (Jiang et al., 2009a). Generally, the freeze-drying method has characteristics of eliminating 95–99% water, obtaining the loose and porous materials, maintaining the original structure and volume, and restraining microbial growth and enzyme function, which is widely used in the pharmaceutical industry, food industry, and biomedical fields. It noted that these are still some problems requiring improvement for this method. Moreover the requirement of expensive equipment, this method is just a means of posttreatment measure. Of course, this method always needs to combine with other synthetic methods. Chong *et al.* (2015) reported the preparation of calcite CaCO_3_-loaded cellulose aerogel for removal of Congo Red (CR) from aqueous solution by freeze-drying. The aerogel had significantly enhanced adsorption capacity toward CR. It obtained the maximum adsorption capacity of 75.81 mg g^−1^ for the CaCO_3_-cellulose aerogel. Narwade *et al.* (2019) used the one-directional freeze-drying technique to obtain flexible and lightweight HA/CNFs nanocomposite films. It observed the detection limit of ammonia at a concentration as low as 5 ppm, sensitivity up to 575%, and response/recovery (210/30s) for nanocomposite films.

The mechanochemical reaction method was also called the high-energy ball milling method. As an energy-saving and efficient technology for the preparation of materials, it significantly reduces the reaction activation energy, refines the grain, and enhances the bonding interface. Yoshida and coworkers (Yoshida et al., 2005) prepared the cellulose/B-type CHA composites through mechanochemical reaction. Then, they applied this method to synthesize cellulose/CHA composites with a bending streng of 10–13 thMPa and Young’s modulus of 1.5–2.2 GPa (Yoshida et al., 2006). In general, the mechanochemical reaction method has the disadvantages of low efficiency and energy consumption, as a supplementary method.

The sonochemical method is a green methodology, which has characteristics of intense local heating, high pressures, and extremely rapid cooling rates (Gedanken, 2004; Bang and Suslick, 2010; Cravotto and Cintas, 2010; Chemat et al., 2011). The ultrasound had wide applications in organic and inorganic synthesis. Stoica-Guzun *et al.* (2012) investigated the CaCO_3_ deposition on BC membranes by ultrasonic irradiation. It obtained the calcite in the presence of ultrasonic irradiation and vaterite in the absence of ultrasonic irradiation. Moreover, it found cubes of calcite to spherical and flower-like vaterite particles in the presence of ultrasonic irradiation. Fu *et al.* did a system study about calcium-containing cellulose-based composites by the ultrasound method. For example, the influences of synthesis strategies of the microwave method and ultrasound method were investigated on the CaCO_3_ in the cellulose matrix (Fu et al., 2013a). It obtained the vaterite spheres with a diameter of about 320–600 nm by the ultrasound method. The CaCO_3_ crystals with good biocompatibility on the cellulose substrate had biomedical applications. The growth mechanism of vaterite was explored on the cellulose matrix via the sonochemistry process (Fu et al., 2013b). It achieved the vaterite polymorph using Na_2_CO_3_ as a reactant in ethylene glycol in the cellulose by the sonochemistry method. Moreover, cellulose/HA nanocomposites with good cytocompatibility were obtained via the sonochemical synthetic method for application in protein adsorption (Fu LH. et al., 2019). It achieved a relatively high protein adsorption ability. Fu *et al.* (2019a) used the sonochemical method to obtain cellulose/vaterite nanospheres with a diameter of 206–246 nm. It found cytocompatibility and a relatively high protein adsorption ability for cellulose/vaterite nanocomposites. Nicoara *et al.* (2020) used the co-precipitation method and ultrasound exposure to *in situ* and *ex situ* design HA/BC/Ag composite with excellent biocompatibility, bioactivity, and antibacterial properties for tissue engineering. It carried out a homogenous porous structure and high water absorption capacity for the composites.

### 1.4 Properties and Applications of Calcium-Containing Cellulose-Based Composites

#### 1.4.1 Properties and Applications of Cellulose/Hydroxyapatite Composites

It has been accepted that cellulose/HA composites are promising bone substitutes. Therefore, it is very important for cellulose/HA composites to have mechanical properties analogous to natural bone. Yoshida *et al.* (2006) synthesized cellulose/CHA composites with good mechanical properties and bioactivity through mechanochemical reactions. Pure CHA had a density of 1.26 g cm^−3^, bending strengths of 5.4 MPa, and Young’s modulus of 1.58 GPa. However, the cellulose/CHA composites displayed a density of 1.59 g cm^−3^, bending strengths of 13.0 MPa, and Young’s modulus of 2.18 GPa. By immersing in SBF for some time, HA with low crystallinity was carried out at the surface of cellulose/CHA composites, displaying good bioactivity. Undoubtedly, all the mechanical properties, bioactivity, and high chemical durability are very important for cellulose/CHA composites to use as bioactive bone substitutes.

As for HA/chitosan/CMC composites, Jiang and coworkers did system research on the mechanical property, swelling behavior, degradation, and bioactivity. Jiang L. Y. et al. (2008) prepared HA/chitosan/CMC composites by the freeze-drying method. It was found that the HA/chitosan/CMC composites with 30 wt% CMC had a pore size of 100–500 μm and porosity of 77.8%, the compressive strength of 3.54 MPa, and bioactivity *in vitro* in the SBF soaking. Then, the HA/chitosan/CMC composites with high bioactivity and adjustable biodegradation rate were carried out by the cosolution method (Jiang et al., 2009b). It achieved the value compressive strength of 85.03 74.91 MPa in the HA/chitosan/CMC composites for weight ratios of 70/15/15, compared with that of HA/chitosan (61.26 MPa). After that, they also prepared HA/chitosan/CMC composite membrane with the highest tensile strength of 40 MPa by self-assembly of static electricity. By soaking in 1.5 SBF, it observed the increased number of apatite particles on the surface of the HA/chitosan/CMC composite membrane. Generally, it is agreed that the HA/chitosan/CMC composites with mechanical properties, swelling behavior, adjustable degradation, and high bioactivity had applications in bone tissue regeneration.

#### 1.4.2 Biological Properties as Tissue Engineering Scaffolds

Grande *et al.* (2009) prepared BC/calcium-deficient HA nanocomposites with biocompatibility and cell viability by a precipitation method. It found the cell viability of 97.2% for HEK cells in the BC/calcium-deficient HA nanocomposites, more than that of BC (86.8%). They suggested that all pore sizes, fiber diameter, and chemical bond of HA in nanocomposites influenced the cell viability of HEK cells. Saska *et al.* (2011) prepared biocompatible BC/HA nanocomposite membranes with biological properties for bone regeneration by *in vivo* tests. At 4 weeks, BC/HA composites displayed newly formed bone with several osteocytes, blood vessels, and bone matrix filled in bone defects. It found HA of low crystallinity with a Ca/P molar rate (1.5) similar to that of physiological bone.

Tazi *et al.* (2012) used BC scaffold to support osteoblast growth and bone formation and used BC/HA membranes to evaluate osteoblast growth. It observed the significantly increased cell growth and spreading on the surface of BC/HA membranes, compared with that of BC. They demonstrated that BC could sustain osteoblast adhesion and the HA enhanced osteoblast adhesion and spreading. Hammonds *et al.* (2012) investigated the feasibility of generating calcium-deficient HA from BC/calcium-deficient HA composites by thermal and enzymatic methods. The degradation method produced calcium-deficient HA, providing an example for the composites as a bone filler.

Tommila *et al.* (2009) found that cellulose sponges coated with HA attracted circulating hemopoietic and mesenchymal progenitor cells efficiently and contained calcium-sensing receptors-positive cells. It probably suggested that the stem cells were responsible for the richly vascularized granulation tissue formed in HA-coated sponges. Liu *et al.* (2010) used HA/polyurethane composite scaffold to generate an antibiotic drug delivery system with good cytocompatibility and antibacterial properties. It found a sustained release of the model drug for up to 60 days Wang *et al.* (2013) prepared a porous BC membrane with gelatin and CHA with low crystallite size and crystallinity via the laser patterning technique. It showed that the C5.18 cells survived after being cultured in the 3D BC scaffolds for 7 days. The chondrogenic rat cell could keep viability on scaffolds, which indicated the scaffolds with good cytocompatibility. It is known that chitosan possessed innate antimicrobial properties toward both Gram-positive and Gram-negative organisms, which could be used in the wound dressings without antimicrobial infections during the implants produce. Mututuvari *et al.* (2013) synthesized cellulose/chitosan/HA composite with good antimicrobial activity.

**FIGURE 3:**
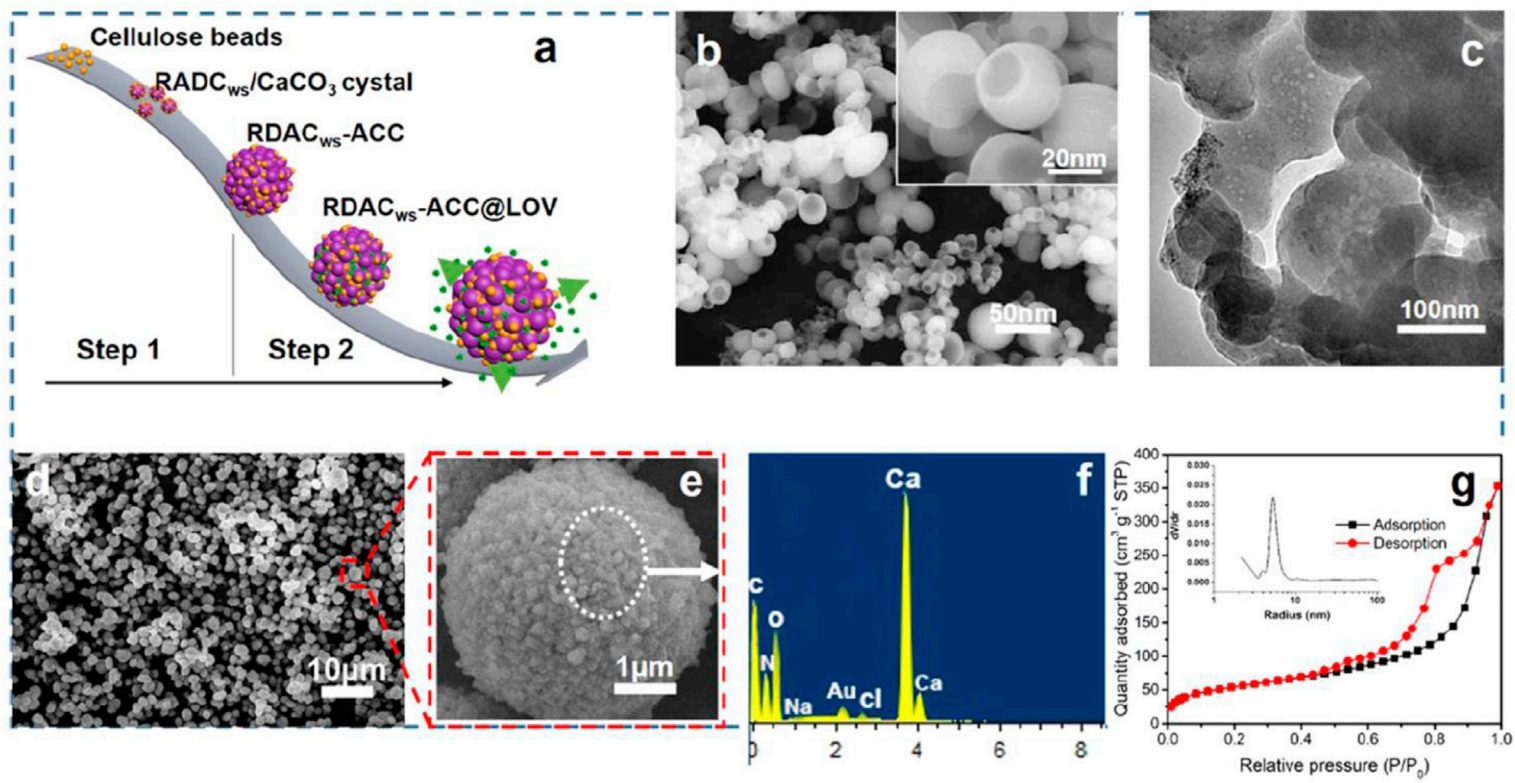
**(A)** Schematic illustration for the preparation of CaCO_3_ microspheres and drug loading and release procedures, **(B)** The scanning electron microscopy (SEM) image of the hollow cellulose-based NPs (DACws after aging for 10 days at 60 °C) and the corresponding magnification diagram (inset), **(C)** the transmission electron microscopy (TEM) image of the hollow cellulose-based NPs, **(D)** the SEM image of CaCO_3_ microspheres with hollow cellulose-based NPs as the template, **(E)** the corresponding enlarged image of the CaCO_3_ microspheres, **(F)** the corresponding energy-dispersive X-ray spectroscopy (EDS) pattern of the particles, and **(G)** the corresponding N_2_ adsorption-desorption isotherm and pore size distribution (inset) of CaCO_3_ microspheres (Yan et al., 2019).

**FIGURE 4:**
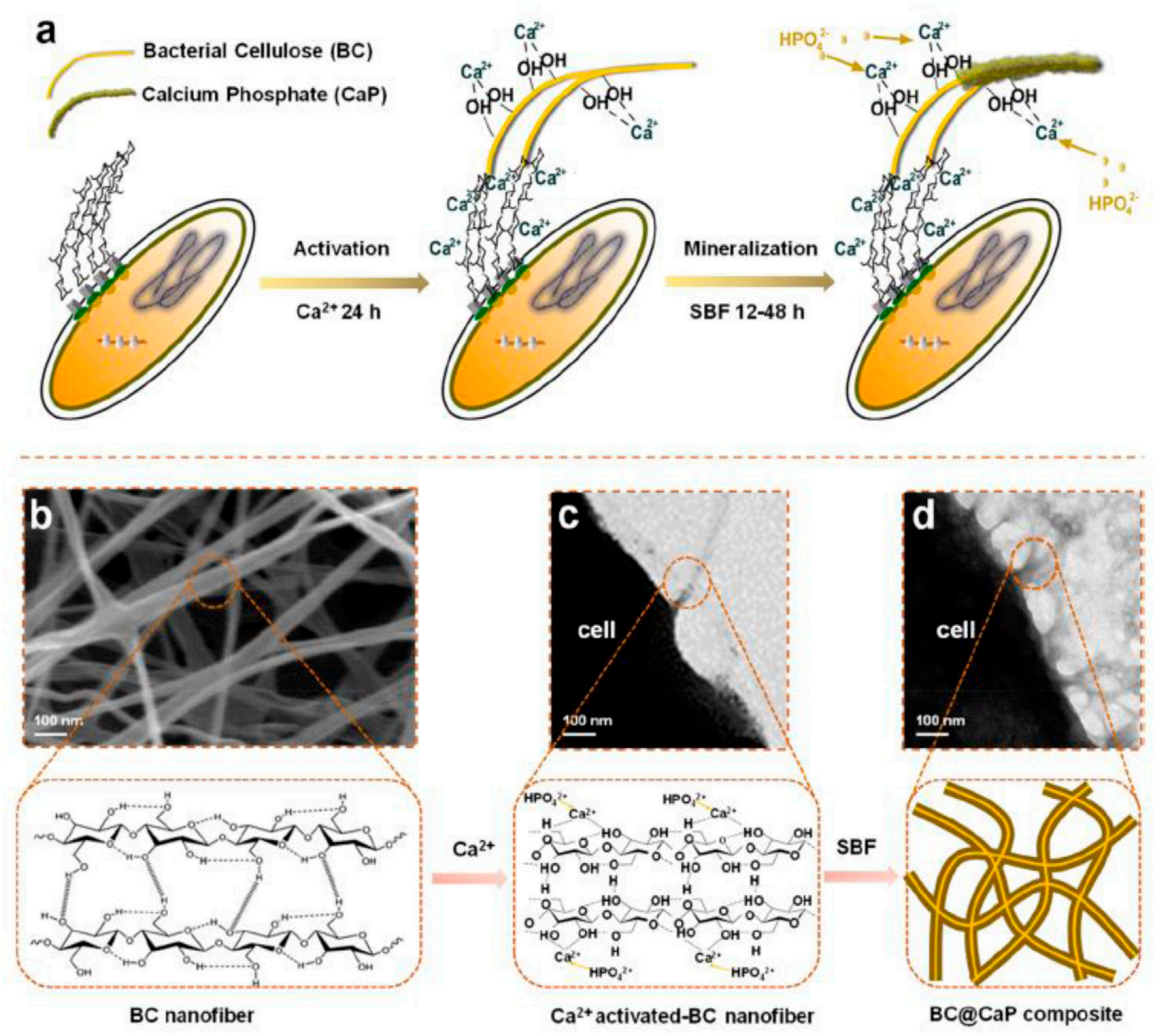
In the experimental design of this study, hydroxyl-rich cellulose molecule chains capture free Ca^2+^ to initiate the nucleation of HAp. **(A)** Schematic diagram of the formation of HAp accompanying the production of cellulose by **(A)** x. **(B)** The representative SEM image of cellulose nanofibers secreted by bacterial cells. **(C)** The representative TEM image of cellulose nanofibers secreted by bacterial cells. **(D)** The representative TEM image of the BC@HAp composite was obtained under biological metabolism conditions. (Chen et al., 2021).

Yan *et al.* (2019) prepared biocompatible dialdehyde cellulose/CaCO_3_ microspheres about 2–3.5 μm and a high specific surface area of similar to 363 m^2^ g^−1^ for tunable pH-responsive drug delivery (Figure 3A). It observed a small and uniform template (Figure 3B) and a porous structure (Figure 3C). It obtained uniform microspheres by hollow cellulose (Figure 3D) formed by the aggregation nanoparticles (Figure 3E). A strong Ca signal was observed (Figure 3F). It carried out a surface area of ∼363.1 m^2^/g and a diameter of 10 nm for CaCO_3_ microspheres (Figure 3G). It found encapsulation efficiency, pH responsiveness, and biocompatibility for the porous microspheres. Chen *et al.* (2021) reported the mineralization of biomimetic HA/nanocellulose nanocomposites with a higher Young modulus. It found the easier capture of Ca^2+^ by the abundant hydroxyl groups on the glucan chain before the formation of hydrogen bonding for the subsequent growth of HA crystals (Figure 4). Gao J. et al. (2022) developed flexible and superhydrophilic ultralong HA nanowire-based biopaper with tensile strength (2.57 MPa), porosity (77%), and specific surface area (36.84 m^2^ g^−1^) for a wound dressing. It found the proliferation, migration, and *in vitro* angiogenesis of HUVECs for the biopaper.

Nasrallah and Ibrahim (2022) reported physico-chemical, dielectric, and antimicrobial properties of the PVA/CMC blend films by silver doped HA nanoparticles using the casting technique. The films had antimicrobial properties for antibacterial activity against Bacillus subtilis, Escherichia coli, and Candida albicans. Nanocomposites had improved electrical conductivity and antimicrobial efficiency by doping silver/HA nanoparticles. Gao F. et al. (2022) prepared deacetylate 3D porous CA/HA/polydopamine microspheres coating with excellent attachment, adhesion, and proliferation as the scaffold for bone tissue regeneration. The microspheres coating was capable of differentiating osteogenically via *in vitro* mineralization.

## 2 Future Perspectives

It is widely known that there are increasing demands in the biomedical fields. The calcium-containing cellulose-based composites included cellulose/HA, cellulose/calcium carbonate, and cellulose/calcium silicate composites in this review study. Obviously, the calcium-containing cellulose-based composites had the characteristics of cellulose and HA/CaCO_3_/CaSiO_3_ and induced some new properties. As aforementioned, the cellulose/HA composites possessed high mechanical properties, good swelling behavior, adjustable biodegradation, high bioactivity, high chemical durability, good cytocompatibility, and excellent ion-exchangeability. Therefore, it is believed that these materials are promising candidates for the applications in the biomedical fields such as bone regeneration, bone tissue engineering, drug delivery, etc. In order to realize the realistic applications, based on the as-reported works in the literature, the following issues need to be explored in the near future.

First, the interaction mechanism between cellulose and inorganic calcium is still unknown and needs to be explored. There are a few reports on the interaction mechanism between cellulose and CaCO_3_. As aforementioned, obviously, there exists a strong interaction between these two apartments. Halab-Kessira and Ricard (1996a), and Halab-Kessira and Ricard (1996b) investigated the interaction of CaCO_3_ particles with cellulose grafted poly acrylate of trimethylaminoethyl chloride (PCMA) copolymers. Experimental results indicated that the retention of calcium carbonate particles was strongly dependent on the cationic content of the copolymer and electro-interactions make the adsorption easier. It obtained the maximum cover of about 750 mg g^−1^ for grafted fiber, and 280 mg g^−1^ for ungrafted fiber. As we all know, it is very important for the composites to display the applications by understanding the surface and interface properties. More importantly, as aforementioned, it showed that apatite particles grow on the surface of the cellulose/HA composites. The surface and interface properties are directly related to biomedical applications. Although some groups suggested the combination between cellulose and HA through hydrogen bonding, obviously, there is still a lack of experimental evidence and theoretical explanation. It assumed that the interaction is the hydrogen bond. The intramolecular hydrogen bonding or intermolecular hydrogen bonding, the strength of the hydrogen bonding, the hydrogen bonding be quantitative or semi-quantitative analysis should be investigated. We would like to know how the hydrogen bonding formed between cellulose and HA and whether the stability of cellulose is enhanced or weakened by hydrogen bonding. In addition to the hydrogen bonding, there exist other interactions such as electrostatic force, van der Waals, etc. Moreover, it is easy to observe the HA crystals dispersed on the surface of cellulose. The formation mechanism should be solved. Therefore, the intrinsic and detailed interaction mechanism between cellulose and HA needs to be further investigated. In fact, the research of mechanism is of great importance for the applications of both the cellulose/HA composites and other composites.

Second, the calcium-containing cellulose-based composites, especially cellulose/HA composites, should be explored for the industrialization application. Although calcium-containing cellulose-based composites are promising biomedical materials, there is a long road ahead for the industrialization application. Undoubtedly, it is a long process for the industrialization process of biomaterials. Obviously, the research on calcium-containing cellulose-based composites is at the initial stage. There are many problems that need to be solved. In comparison with other biomedical materials, the advantages of calcium-containing cellulose-based composites should be highlighted. For example, at present, there is a lack of comparative study of cellulose/HA composites and other biomedical materials. We should pay attention to the comparative study in the next stage. In addition, more biomedical properties including protein adsorption, gene carrier, adsorption/release, the performance of bone repair, and vascular properties should be explored.

Third, cellulose itself is certainly worthy of more attention. It reports that cellulose is extracted from sources including wood, plant, tunicate, algae, bacterial, etc. There are four polymorphs of crystalline cellulose (I, II, III, IV), which consist of crystalline and amorphous regions. Each polymorph has a different crystalline structure. The cellulose was extracted from cellulose sources via the mechanical treatment, acid hydrolysis, and enzymatic hydrolysis method by the complete or partial removal of matrixes (hemicellulose, lignin, etc.). In addition, it reports that there are nine particle types of cellulose. Each particle type has a characteristic size, morphology, crystallinity, and properties. Therefore, choosing an appropriate type of cellulose is important for the formation and applications of calcium-containing cellulose-based composites. For example, the existence of hemicellulose and lignin on the properties of composites should be researched.

As aforementioned, there are many methods for the synthesis of calcium-containing cellulose-based composites. Much attention has been paid to the biomineralization method. Indeed, HA is close to the natural bone in the cellulose/HA composites by the biomineralization method. This is a good method to investigate the mechanism of biomineralization. However, this method has the disadvantages of being time-consuming, low-productivity, and poor reproducibility. Obviously, it is not the best choice for the industrialization process of biomaterials. Moreover, the synthesis method should meet the principles of environment friendliness, economic friendliness, low cost, and high yield.

Finally, the calcium-containing cellulose-based composites itself is certainly worthy of more attention. As described in the literature, it is believed that the composites with specific porous structures could produce bone activity and osteoblast cells could have good adhesion and proliferation at the surface of composites (Yuan et al., 2010). It is reported that the histological evidence on the special micro or nanostructure induces bone regeneration (Zhu et al., 2009). It is well known that both cellulose and inorganic calcium have various morphologies and structures. Therefore, more attention should be paid to the pore and microstructure of calcium-containing cellulose-based composites. For example, cellulose/HA composites with 3D patterning should be fabricated by the electrospinning method and could be processed into film or bio-ceramic. Moreover, it is found that ions could promote cell proliferation and activate the gene expression (Lin et al., 2011). Nature HA also includes the chemical compositions of Na, Mg, Sr, Si, K, F, Cl, and CO_3_^2-^, which favored the improved biological properties of HA. The cellulose/HA composites with other chemical compositions should also be prepared.

## 3 Conclusion

In this mini-review article, we described the recent advances in the synthesis, properties, and biomedical applications of calcium-containing cellulose-based composites including cellulose/HA, cellulose/calcium carbonate, and cellulose/calcium silicate composites. The future developments and applications of calcium-containing cellulose-based composites were given. It expects that more attention is paid to the research of calcium-containing cellulose-based composites.

## References

[B1] Addadi L. Raz S. Weiner S. (2003). Taking Advantage of Disorder: Amorphous Calcium Carbonate and its Roles in Biomineralization. Adv. Mat. 15 (12), 959–970. 10.1002/adma.200300381 10.1002/adma.200300381 | Google Scholar

[B2] Baghbanzadeh M. Carbone L. Cozzoli P. D. Kappe C. O. (2011). Microwave-assisted Synthesis of Colloidal Inorganic Nanocrystals. Angew. Chem. Int. Ed. 50, 11312–11359. 10.1002/anie.201101274 10.1002/anie.201101274 | Google Scholar 22058070

[B3] Bang J. H. Suslick K. S. (2010). Applications of Ultrasound to the Synthesis of Nanostructured Materials. Adv. Mat. 22, 1039–1059. 10.1002/adma.200904093 PubMed Abstract | 10.1002/adma.200904093 | Google Scholar 20401929

[B4] Byrappa K. Adschiri T. (2007). Hydrothermal Technology for Nanotechnology. Prog. Cryst. Growth Charact. Mater. 53, 117–166. 10.1016/j.pcrysgrow.2007.04.001 10.1016/j.pcrysgrow.2007.04.001 | Google Scholar

[B5] Chan C.-M. Wu J. Li J.-X. Cheung Y.-K. (2002). Polypropylene/calcium Carbonate Nanocomposites. Polymer 43, 2981–2992. 10.1016/s0032-3861(02)00120-9 10.1016/s0032-3861(02)00120-9 | Google Scholar

[B6] Chemat F. Zill-E-Huma Khan M. K. (2011). Applications of Ultrasound in Food Technology: Processing, Preservation and Extraction. Ultrason. Sonochemistry 18, 813–835. 10.1016/j.ultsonch.2010.11.023 PubMed Abstract | 10.1016/j.ultsonch.2010.11.023 | Google Scholar 21216174

[B7] Chen C. Qian J. Chen H. Zhang H. Yang L. Jiang X. (2021). Molecular Origin of the Biologically Accelerated Mineralization of Hydroxyapatite on Bacterial Cellulose for More Robust Nanocomposites. Nano Lett. 21, 10292–10300. 10.1021/acs.nanolett.1c03411 PubMed Abstract | 10.1021/acs.nanolett.1c03411 | Google Scholar 34846904

[B8] Cheng X. F. Qian H. Zhang S. W. Zhang Z. S. He Y. Ma M. G. (2016). Preparation and Characterization of Cellulose-CaCO_3_ Composites by an Eco-Friendly Microwave-Assisted Route in a Mixed Solution of Ionic Liquid and Ethylene Glycol. Bioresources 11, 4392–4401. 10.15376/biores.11.2.4392-4401 10.15376/biores.11.2.4392-4401 | Google Scholar

[B9] Chong K. Y. Chia C. H. Zakaria S. Sajab M. S. Chook S. W. Khiew P. S. (2015). CaCO3-decorated Cellulose Aerogel for Removal of Congo Red from Aqueous Solution. Cellulose 22, 2683–2691. 10.1007/s10570-015-0675-2 10.1007/s10570-015-0675-2 | Google Scholar

[B10] Chu T.-M. G. Orton D. G. Hollister S. J. Feinberg S. E. Halloran J. W. (2002). Mechanical and *In Vivo* Performance of Hydroxyapatite Implants with Controlled Architectures. Biomaterials 23, 1283–1293. 10.1016/s0142-9612(01)00243-5 PubMed Abstract | 10.1016/s0142-9612(01)00243-5 | Google Scholar 11808536

[B11] Ciobanu M. Bobu E. Ciolacu F. (2010). *In-situ* Cellulose Fibres Loading with Calcium Carbonate Precipitated by Different Methods. Cellul. Chem. Technol. 44, 379–387. Google Scholar

[B12] Cravotto G. Cintas P. (2010). Power Ultrasound in Organic Synthesis: Moving Cavitational Chemistry from Academia to Innovative and Large-Scale Applications. Chem. Soc. Rev. 35, 180–196. 10.1039/b503848k 10.1039/b503848k | Google Scholar 16444299

[B13] Cromme P. Zollfrank C. Müller L. Müller F. A. Greil P. (2007). Biomimetic Mineralisation of Apatites on Ca2+ Activated Cellulose Templates. Mater. Sci. Eng. C 27, 1–7. 10.1016/j.msec.2005.11.001 10.1016/j.msec.2005.11.001 | Google Scholar

[B14] Cüneyt Tas A. (2000). Synthesis of Biomimetic Ca-Hydroxyapatite Powders at 37°C in Synthetic Body Fluids. Biomaterials 21, 1429–1438. 10.1016/s0142-9612(00)00019-3 PubMed Abstract | 10.1016/s0142-9612(00)00019-3 | Google Scholar 10872772

[B15] Doerner H. A. Hoskins W. M. (1925). Co-Precipitation of Radium and Barium Sulfates1. J. Am. Chem. Soc. 47, 662–675. 10.1021/ja01680a010 10.1021/ja01680a010 | Google Scholar

[B16] Dorozhkin S. V. (2011). Calcium Orthophosphates. Biomatter 1, 121–164. 10.4161/biom.18790 PubMed Abstract | 10.4161/biom.18790 | Google Scholar 23507744 PMC3549886

[B17] Eichhorn S. J. Dufresne A. Aranguren M. Marcovich N. E. Capadona J. R. Rowan S. J. (2010). Review: Current International Research into Cellulose Nanofibres and Nanocomposites. J. Mat. Sci. 45, 1–33. 10.1007/s10853-009-3874-0 10.1007/s10853-009-3874-0 | Google Scholar PMC7307936 32836382

[B18] Fang B. Wan Y.-Z. Tang T.-T. Gao C. Dai K.-R. (2009). Proliferation and Osteoblastic Differentiation of Human Bone Marrow Stromal Cells on Hydroxyapatite/bacterial Cellulose Nanocomposite Scaffolds. Tissue Eng. Part A 15, 1091–1098. 10.1089/ten.tea.2008.0110 PubMed Abstract | 10.1089/ten.tea.2008.0110 | Google Scholar 19196148

[B19] Feng S. Xu R. (2001). New Materials in Hydrothermal Synthesis. Acc. Chem. Res. 34, 239–247. 10.1021/ar0000105 PubMed Abstract | 10.1021/ar0000105 | Google Scholar 11263882

[B20] Fu L.-H. Dong Y.-Y. Ma M.-G. Li S.-M. Sun R.-C. (2013a). Compare Study CaCO3 Crystals on the Cellulose Substrate by Microwave-Assisted Method and Ultrasound Agitation Method. Ultrason. Sonochemistry 20, 839–845. 10.1016/j.ultsonch.2012.11.001 PubMed Abstract | 10.1016/j.ultsonch.2012.11.001 | Google Scholar 23200085

[B21] Fu L.-H. Dong Y.-Y. Ma M.-G. Yue W. Sun S.-L. Sun R.-C. (2013b). Why to Synthesize Vaterite Polymorph of Calcium Carbonate on the Cellulose Matrix via Sonochemistry Process? Ultrason. Sonochemistry 20, 1188–1193. 10.1016/j.ultsonch.2013.03.008 10.1016/j.ultsonch.2013.03.008 | Google Scholar 23591018

[B22] Fu L.-H. Liu Y.-J. Ma M.-G. Zhang X.-M. Xue Z.-M. Zhu J.-F. (2016). Microwave-assisted Hydrothermal Synthesis of Cellulose/hydroxyapatite Nanocomposites. Polymers 8, 316. 10.3390/polym8090316 PubMed Abstract | 10.3390/polym8090316 | Google Scholar PMC6432507 30974621

[B23] Fu L.-H. Qi C. Hu Y.-R. Mei C.-G. Ma M.-G. (2019a). Cellulose/vaterite Nanocomposites: Sonochemical Synthesis, Characterization, and Their Application in Protein Adsorption. Mater. Sci. Eng. C 96, 426–435. 10.1016/j.msec.2018.11.061 PubMed Abstract | 10.1016/j.msec.2018.11.061 | Google Scholar 30606552

[B24] Fu L.-H. Qi C. Ma M.-G. Wan P. (2019c). Multifunctional Cellulose-Based Hydrogels for Biomedical Applications. J. Mat. Chem. B 7, 1541–1562. 10.1039/c8tb02331j 10.1039/c8tb02331j | Google Scholar 32254901

[B25] Fu L.-H. Xie Y.-M. Bian J. Ma M.-G. Tian C.-H. Jin X.-J. (2015). Microwave-assisted Rapid Synthesis of Lignocellulose/hydroxyapatite Nanocomposites. Mater. Lett. 159, 51–53. 10.1016/j.matlet.2015.06.082 10.1016/j.matlet.2015.06.082 | Google Scholar

[B26] Fu L. H. Qi C. Liu Y. J. Cao W. T. Ma M. G. (2019b). Sonochemical Synthesis of Cellulose/hydroxyapatite Nanocomposites and Their Application in Protein Adsorption. Sci. Rep. 8, 8292. 10.1038/s41598-018-25566-7 PubMed Abstract | 10.1038/s41598-018-25566-7 | Google Scholar PMC5974341 29844448

[B27] Gao F. Zeng D. Liu H. Qin R. Zhang J. Chen Y. (2022a). Porous Cellulose Microspheres Coated in One Step with a Polydopamine Suspension of Hydroxyapatite for Bone Tissue Engineering. Cellulose 29, 1955–1967. 10.1007/s10570-021-04395-4 10.1007/s10570-021-04395-4 | Google Scholar

[B28] Gao J. Hao L.-S. Ning B.-B. Zhu Y.-K. Guan J.-B. Ren H.-W. (2022b). Biopaper Based on Ultralong Hydroxyapatite Nanowires and Cellulose Fibers Promotes Skin Wound Healing by Inducing Angiogenesis. Coatings 12, 479. 10.3390/coatings12040479 10.3390/coatings12040479 | Google Scholar

[B29] Garai S. Sinha A. (2014). Biomimetic Nanocomposites of Carboxymethyl Cellulose-Hydroxyapatite: Novel Three Dimensional Load Bearing Bone Grafts. Colloids Surfaces B Biointerfaces 115, 182–190. 10.1016/j.colsurfb.2013.11.042 PubMed Abstract | 10.1016/j.colsurfb.2013.11.042 | Google Scholar 24342800

[B30] Garrote G. Domínguez H. Parajó J. C. (1999). Hydrothermal Processing of Lignocellulosic Materials. Holz als Roh- Werkst. 57, 191–202. 10.1007/s001070050039 10.1007/s001070050039 | Google Scholar

[B31] Gedanken A. (2004). Using Sonochemistry for the Fabrication of Nanomaterials. Ultrason. Sonochemistry 11, 47–55. 10.1016/j.ultsonch.2004.01.037 10.1016/j.ultsonch.2004.01.037 | Google Scholar 15030779

[B32] Gibson I. R. Bonfield W. (2002). Novel Synthesis and Characterization of an AB-type Carbonate-Substituted Hydroxyapatite. J. Biomed. Mat. Res. 59, 697–708. 10.1002/jbm.10044 PubMed Abstract | 10.1002/jbm.10044 | Google Scholar 11774332

[B33] Gower L. B. (2008). Biomimetic Model Systems for Investigating the Amorphous Precursor Pathway and its Role in Biomineralization. Chem. Rev. 108, 4551–4627. 10.1021/cr800443h PubMed Abstract | 10.1021/cr800443h | Google Scholar 19006398 PMC3652400

[B34] Grande C. J. Torres F. G. Gomez C. M. Carmen Bañó M. (2009). Nanocomposites of Bacterial Cellulose/hydroxyapatite for Biomedical Applications. Acta Biomater. 5, 1605–1615. 10.1016/j.actbio.2009.01.022 PubMed Abstract | 10.1016/j.actbio.2009.01.022 | Google Scholar 19246264

[B35] Habraken W. Habibovic P. Epple M. Bohner M. (2016). Calcium Phosphates in Biomedical Applications: Materials for the Future? Mater. Today 19, 69–87. 10.1016/j.mattod.2015.10.008 10.1016/j.mattod.2015.10.008 | Google Scholar

[B36] Halab-Kessira L. Ricard A. (1996b). Adsorption of CaCO3Particles on Cationic Cellulose Graft Copolymers. J. Colloid Interface Sci. 184, 437–442. 10.1006/jcis.1996.0638 PubMed Abstract | 10.1006/jcis.1996.0638 | Google Scholar 8978546

[B37] Halab-Kessira L. Ricard A. (1996a). Adsorption of CaCO3Particles on Cationic Cellulose Graft Copolymers I Effect of Chemical Parameters. J. Colloid Interface Sci. 179, 269–275. 10.1006/jcis.1996.0213 PubMed Abstract | 10.1006/jcis.1996.0213 | Google Scholar 8978546

[B38] Hammonds R. L. Harrison M. S. Cravanas T. C. Gazzola W. H. Stephens C. P. Benson R. S. (2012). Biomimetic Hydroxyapatite Powder from a Bacterial Cellulose Scaffold. Cellulose 19, 1923–1932. 10.1007/s10570-012-9767-4 10.1007/s10570-012-9767-4 | Google Scholar

[B39] Hofmann I. Müller L. Greil P. Müller F. A. (2006). Calcium Phosphate Nucleation on Cellulose Fabrics. Surf. Coatings Technol. 201, 2392–2398. 10.1016/j.surfcoat.2006.04.007 10.1016/j.surfcoat.2006.04.007 | Google Scholar

[B40] Hokkanen S. Bhatnagar A. Repo E. Lou S. Sillanpää M. (2016). Calcium Hydroxyapatite Microfibrillated Cellulose Composite as a Potential Adsorbent for the Removal of Cr(VI) from Aqueous Solution. Chem. Eng. J. 283, 445–452. 10.1016/j.cej.2015.07.035 10.1016/j.cej.2015.07.035 | Google Scholar

[B41] Hong L. Wang Y. L. Jia S. R. Huang Y. Gao C. Wan Y. Z. (2006). Hydroxyapatite/bacterial Cellulose Composites Synthesized via a Biomimetic Route. Mater. Lett. 60, 1710–1713. 10.1016/j.matlet.2005.12.004 10.1016/j.matlet.2005.12.004 | Google Scholar

[B42] Hu F. Xu S. Liu B. (2018). Photosensitizers with Aggregation-Induced Emission: Materials and Biomedical Applications. Adv. Mat. 30, 1801350. 10.1002/adma.201801350 PubMed Abstract | 10.1002/adma.201801350 | Google Scholar 30066341

[B43] Huang C. Dong H. Zhang Z. Bian H. Yong Q. (2020). Procuring the Nano-Scale Lignin in Prehydrolyzate as Ingredient to Prepare Cellulose Nanofibril Composite Film with Multiple Functions. Cellulose 27, 9355–9370. 10.1007/s10570-020-03427-9 10.1007/s10570-020-03427-9 | Google Scholar

[B44] Huang C. Xu C. Meng X. Wang L. Zhou X. (2022). Editorial: isolation, modification, and characterization of the constituents (cellulose, hemicellulose, lignin, et al.) in biomass and their bio-based applications. Front. Bioeng. Biotechnol. 10, 866531. 10.3389/fbioe.2022.866531 PubMed Abstract | 10.3389/fbioe.2022.866531 | Google Scholar 35646857 PMC9136403

[B45] Hutchens S. A. Benson R. S. Evans B. R. O'Neill H. M. Rawn C. J. (2006). Biomimetic Synthesis of Calcium-Deficient Hydroxyapatite in a Natural Hydrogel. Biomaterials 27, 4661–4670. 10.1016/j.biomaterials.2006.04.032 PubMed Abstract | 10.1016/j.biomaterials.2006.04.032 | Google Scholar 16713623

[B46] Jia N. Li S.-M. Ma M.-G. Sun R.-C. (2011a). Microwave-assisted Ionic Liquid Preparation and Characterization of Cellulose/calcium Silicate Nanocomposites in Ethylene Glycol. Mater. Lett. 65, 918–921. 10.1016/j.matlet.2010.12.033 10.1016/j.matlet.2010.12.033 | Google Scholar

[B47] Jia N. Li S.-M. Ma M.-G. Sun R.-C. (2012a). Rapid Microwave-Assisted Fabrication of cellulose/F-Substituted Hydroxyapatite Nanocomposites Using Green Ionic Liquids as Additive. Mater. Lett. 68, 44–46. 10.1016/j.matlet.2011.10.027 10.1016/j.matlet.2011.10.027 | Google Scholar

[B48] Jia N. Li S.-M. Ma M.-G. Sun R.-C. Zhu J.-F. (2012b). Hydrothermal Fabrication, Characterization, and Biological Activity of cellulose/CaCO3 Bionanocomposites. Carbohydr. Polym. 88, 179–184. 10.1016/j.carbpol.2011.11.086 10.1016/j.carbpol.2011.11.086 | Google Scholar

[B49] Jia N. Li S.-M. Ma M.-G. Sun R.-C. Zhu J.-F. (2010a). Hydrothermal Synthesis and Characterization of Cellulose-Carbonated Hydroxyapatite Nanocomposites in NaOH-Urea Aqueous Solution. Sci. Adv. Mat. 2, 210–214. 10.1166/sam.2010.1086 10.1166/sam.2010.1086 | Google Scholar

[B50] Jia N. Li S.-M. Ma M.-G. Sun R.-C. Zhu L. (2011b). Green Microwave-Assisted Synthesis of Cellulose/calcium Silicate Nanocomposites in Ionic Liquids and Recycled Ionic Liquids. Carbohydr. Res. 346, 2970–2974. 10.1016/j.carres.2011.10.006 PubMed Abstract | 10.1016/j.carres.2011.10.006 | Google Scholar 22055813

[B51] Jia N. Li S.-M. Zhu J.-F. Ma M.-G. Xu F. Wang B. (2010b). Microwave-assisted Synthesis and Characterization of Cellulose-Carbonated Hydroxyapatite Nanocomposites in NaOH-Urea Aqueous Solution. Mater. Lett. 64, 2223–2225. 10.1016/j.matlet.2010.07.029 10.1016/j.matlet.2010.07.029 | Google Scholar

[B52] Jiang D. Zhang J. (2009). Calcium Phosphate with Well Controlled Nanostructure for Tissue Engineering. Curr. Appl. Phys. 9, S252–S256. 10.1016/j.cap.2009.01.029 10.1016/j.cap.2009.01.029 | Google Scholar

[B53] Jiang L.-Y. Li Y. B. Zhang L. Wang X. J. (2008c). Study on Nano-hydroxyapatite/Chitosan-Carboxymethyl Cellulose Composite Scaffold. J. Inure. Mater. 23, 135–140. 10.3724/sp.j.1077.2008.00135 10.3724/sp.j.1077.2008.00135 | Google Scholar

[B54] Jiang L. Li Y. Wang X. Zhang L. Wen J. Gong M. (2008a). Preparation and Properties of Nano-Hydroxyapatite/chitosan/carboxymethyl Cellulose Composite Scaffold. Carbohydr. Polym. 74, 680–684. 10.1016/j.carbpol.2008.04.035 10.1016/j.carbpol.2008.04.035 | Google Scholar

[B55] Jiang L. Y. Li Y. B. Xiong C. D. (2009a). A Novel Composite Membrane of Chitosan-Carboxymethyl Cellulose Polyelectrolyte Complex Membrane Filled with Nano-Hydroxyapatite . Preparation and Properties. J. Mat. Sci. Mat. Medic. 20, 1645–1652. Google Scholar 10.1007/s10856-009-3720-6 19301105

[B56] Jiang L. Y. Li Y. B. Xiong C. D. (2009b). Preparation and Biological Properties of a Novel Composite Scaffold of Nano-Hydroxyapatite/chitosan/carboxymethyl Cellulose for Bone Tissue Engineering. J. Biomed. Sci. 16, 65. Google Scholar 19594953 10.1186/1423-0127-16-65 PMC2720940

[B57] Jiang L. Y. Li Y. B. Zhang L. Liao J. G. (2008b). Preparation and Properties of a Novel Bone Repair Composite: Nano-Hydroxyapatite/chitosan/carboxymethyl Cellulose. J. Mat. Sci. Mat. Medic. 19, 981–987. Google Scholar 10.1007/s10856-007-3208-1 17665104

[B58] Kang S. Zhao K. Yu D. G. Zheng X. Huang C. (2022). Advances in Biosensing and Environmental Monitoring Based on Electrospun Nanofibers. Adv. Fiber Mat. 4, 404–435. 10.1007/s42765-021-00129-0 10.1007/s42765-021-00129-0 | Google Scholar

[B59] Kaur G. Adhikari R. Cass P. Bown M. Gunatillake P. (2015). Electrically Conductive Polymers and Composites for Biomedical Applications. RSC Adv. 5, 37553–37567. 10.1039/c5ra01851j 10.1039/c5ra01851j | Google Scholar

[B60] Kim M. Yeo M. Kim M. Kim G. (2018). Biomimetic Cellulose/calcium-Deficient-Hydroxyapatite Composite Scaffolds Fabricated Using an Electric Field for Bone Tissue Engineering. RSC Adv. 8, 20637–20647. 10.1039/c8ra03657h PubMed Abstract | 10.1039/c8ra03657h | Google Scholar 35542321 PMC9080802

[B61] Kumar A. P. Mohaideen K. K. Alariqi S. A. S. Singh R. P. (2010). Preparation and Characterization of Bioceramic Nanocomposites Based on Hydroxyapatite (HA) and Carboxymethyl Cellulose (CMC). Macromol. Res. 18, 1160–1167. 10.1007/s13233-010-1208-3 10.1007/s13233-010-1208-3 | Google Scholar

[B62] Landi E. Celotti G. Logroscino G. Tampieri A. (2003). Carbonated Hydroxyapatite as Bone Substitute. J. Eur. Ceram. Soc. 23, 2931–2937. 10.1016/s0955-2219(03)00304-2 10.1016/s0955-2219(03)00304-2 | Google Scholar

[B63] Li K. Wang J. Liu X. Xiong X. Liu H. (2012). Biomimetic Growth of Hydroxyapatite on Phosphorylated Electrospun Cellulose Nanofibers. Carbohydr. Polym. 90, 1573–1581. 10.1016/j.carbpol.2012.07.033 PubMed Abstract | 10.1016/j.carbpol.2012.07.033 | Google Scholar 22944418

[B64] Li L. Zhu Y.-J. Ma M.-G. (2008). Microwave-assisted Preparation of Calcium Sulfate Nanowires. Mater. Lett. 62, 4552–4554. 10.1016/j.matlet.2008.08.040 10.1016/j.matlet.2008.08.040 | Google Scholar

[B65] Li S.-M. Jia N. Zhu J.-F. Ma M.-G. Sun R.-C. (2010). Synthesis of Cellulose-Calcium Silicate Nanocomposites in Ethanol/water Mixed Solvents and Their Characterization. Carbohydr. Polym. 80, 270–275. 10.1016/j.carbpol.2009.11.024 10.1016/j.carbpol.2009.11.024 | Google Scholar

[B66] Li W. Wu P. (2009). Biomimetic Synthesis of Monodisperse Rosette-like Calcite Mesocrystals Regulated by Carboxymethyl Cellulose and the Proposed Mechanism : An Unconventional Rhombohedra-Stacking Route. CrystEngComm 11, 2466–2474. 10.1039/b901580a 10.1039/b901580a | Google Scholar

[B67] Lin K. Chang J. Liu X. Chen L. Zhou Y. (2011). Synthesis of Element-Substituted Hydroxyapatite with Controllable Morphology and Chemical Composition Using Calcium Silicate as Precursor. CrystEngComm 13, 4850–4855. 10.1039/c0ce00835d 10.1039/c0ce00835d | Google Scholar

[B68] Liu H. Zhang L. Shi P. Zou Q. Zuo Y. Li Y. (2010). Hydroxyapatite/polyurethane Scaffold Incorporated with Drug-Loaded Ethyl Cellulose Microspheres for Bone Regeneration. J. Biomed. Mat. Res. 95B, 36–46. 10.1002/jbm.b.31680 PubMed Abstract | 10.1002/jbm.b.31680 | Google Scholar 20665683

[B69] Liu L. He D. Wang G.-S. Yu S.-H. (2011). Bioinspired Crystallization of CaCO3 Coatings on Electrospun Cellulose Acetate Fiber Scaffolds and Corresponding CaCO3 Microtube Networks. Langmuir 27, 7199–7206. 10.1021/la200738n PubMed Abstract | 10.1021/la200738n | Google Scholar 21534560

[B70] Liu W. Du H. Zhang M. Liu K. Liu H. Xie H. (2020). Bacterial Cellulose-Based Composite Scaffolds for Biomedical Applications: A Review. ACS Sustain. Chem. Eng. 8, 7536–7562. 10.1021/acssuschemeng.0c00125 10.1021/acssuschemeng.0c00125 | Google Scholar

[B71] Liu X. Li K. Wu C. Li Z. Wu B. Duan X. (2019). Biomimetic Assembly of Multilevel Hydroxyapatite Using Bacterial Cellulose Hydrogel as a Reactor. CrystEngComm 21, 4859–4863. 10.1039/c9ce01086f 10.1039/c9ce01086f | Google Scholar

[B72] Liu X. Ma Y. Zhou Y. Pei C. Yin G. (2013). A Promising Hybrid Scaffold Material: Bacterial Cellulose *In-Situ* Assembling Biomimetic Lamellar CaCO3. Mater. Lett. 102-103, 91–93. 10.1016/j.matlet.2013.03.121 10.1016/j.matlet.2013.03.121 | Google Scholar

[B73] Lukasheva N. V. Tolmachev D. A. (2016). Cellulose Nanofibrils and Mechanism of Their Mineralization in Biomimetic Synthesis of Hydroxyapatite/native Bacterial Cellulose Nanocomposites: Molecular Dynamics Simulations. Langmuir 32, 125–134. 10.1021/acs.langmuir.5b03953 PubMed Abstract | 10.1021/acs.langmuir.5b03953 | Google Scholar 26652774

[B74] Ma M.-G. Dong Y.-Y. Fu L.-H. Li S.-M. Sun R.-C. (2013). Cellulose/CaCO3 Nanocomposites: Microwave Ionic Liquid Synthesis, Characterization, and Biological Activity. Carbohydr. Polym. 92, 1669–1676. 10.1016/j.carbpol.2012.11.034 PubMed Abstract | 10.1016/j.carbpol.2012.11.034 | Google Scholar 23399205

[B75] Ma M.-G. Fu L.-H. Li S.-M. Zhang X.-M. Sun R.-C. Dai Y.-D. (2012b). Hydrothermal Synthesis and Characterization of Wood powder/CaCO3 Composites. Carbohydr. Polym. 88, 1470–1475. 10.1016/j.carbpol.2012.02.043 10.1016/j.carbpol.2012.02.043 | Google Scholar

[B76] Ma M.-G. Fu L.-H. Sun R.-C. Jia N. (2012a). Compared Study on the cellulose/CaCO3 Composites via Microwave-Assisted Method Using Different Cellulose Types. Carbohydr. Polym. 90, 309–315. 10.1016/j.carbpol.2012.05.043 PubMed Abstract | 10.1016/j.carbpol.2012.05.043 | Google Scholar 24751046

[B77] Ma M.-G. (2012). Hierarchically Nanostructured Hydroxyapatite: Hydrothermal Synthesis, Morphology Control, Growth Mechanism, and Biological Activity. Ijn 7, 1781–1791. 10.2147/ijn.s29884 PubMed Abstract | 10.2147/ijn.s29884 | Google Scholar 22619527 PMC3356187

[B78] Ma M.-G. Zhu J.-F. Jia N. Li S.-M. Sun R.-C. Cao S.-W. (2010). Rapid Microwave-Assisted Synthesis and Characterization of Cellulose-Hydroxyapatite Nanocomposites in N,N-dimethylacetamide Solvent. Carbohydr. Res. 345, 1046–1050. 10.1016/j.carres.2010.03.004 PubMed Abstract | 10.1016/j.carres.2010.03.004 | Google Scholar 20381016

[B79] Ma M.-G. Zhu J.-F. (2010). Recent Progress on Fabrication of Calcium-Based Inorganic Biodegradable Nanomaterials. Nanotec 4, 164–170. 10.2174/187221010792483690 PubMed Abstract | 10.2174/187221010792483690 | Google Scholar 20707769

[B80] Ma M.-G. Zhu J.-F. Zhu Y.-J. Sun R.-C. (2014). The Microwave-Assisted Ionic-Liquid Method: A Promising Methodology in Nanomaterials. Chem. Asian J. 9, 2378–2391. 10.1002/asia.201402288 PubMed Abstract | 10.1002/asia.201402288 | Google Scholar 24895207

[B81] Ma M.-G. Zhu Y.-J. Chang J. (2006). Monetite Formed in Mixed Solvents of Water and Ethylene Glycol and its Transformation to Hydroxyapatite. J. Phys. Chem. B 110, 14226–14230. 10.1021/jp061738r PubMed Abstract | 10.1021/jp061738r | Google Scholar 16854124

[B82] Ma M. G. Jia N. Li S. M. Sun R. C. (2011). Nanocomposites of Cellulose/carbonated Hydroxyapatite by Microwave-Assisted Fabrication in Ionic Liquid: Characterization and Thermal Stability. Iran. Polym. J. 20, 413–421. Google Scholar

[B83] Ma M. G. Zhu J. F. (2009). Solvothermal Synthesis and Characterization of Hierarchically Nanostructured Hydroxyapatite Hollow Spheres. Eur. J. Inorg. Chem. 2009, 5522–5526. 10.1002/ejic.200900839 10.1002/ejic.200900839 | Google Scholar

[B84] Meng L.-Y. Wang B. Ma M.-G. Lin K.-L. (2016). The Progress of Microwave-Assisted Hydrothermal Method in the Synthesis of Functional Nanomaterials. Mater. Today Chem. 1-2, 63–83. 10.1016/j.mtchem.2016.11.003 10.1016/j.mtchem.2016.11.003 | Google Scholar

[B85] Moon R. J. Martini A. Nairn J. Simonsen J. Youngblood J. (2011). Cellulose Nanomaterials Review: Structure, Properties and Nanocomposites. Chem. Soc. Rev. 40, 3941–3994. 10.1039/c0cs00108b PubMed Abstract | 10.1039/c0cs00108b | Google Scholar 21566801

[B86] Morales-Nieto V. Navarro C. H. Moreno K. J. Arizmendi-Morquecho A. Chávez-Valdez A. García-Miranda S. (2013). Poly(methyl Methacrylate)/carbonated Hydroxyapatite Composite Applied as Coating on Ultra High Molecular Weight Polyethylene. Prog. Org. Coatings 76, 204–208. 10.1016/j.porgcoat.2012.09.007 10.1016/j.porgcoat.2012.09.007 | Google Scholar

[B87] Mututuvari T. M. Harkins A. L. Tran C. D. (2013). Facile Synthesis, Characterization, and Antimicrobial Activity of Cellulose-Chitosan-Hydroxyapatite Composite Material: a Potential Material for Bone Tissue Engineering. J. Biomed. Mater Res. A 101, 3266–3277. 10.1002/jbm.a.34636 PubMed Abstract | 10.1002/jbm.a.34636 | Google Scholar 23595871 PMC3788024

[B88] Narwade V. N. Anjum S. R. Kokol V. Khairnar R. S. (2019). Ammonia-sensing Ability of Differently Structured Hydroxyapatite Blended Cellulose Nanofibril Composite Films. Cellulose 26, 3325–3337. 10.1007/s10570-019-02299-y 10.1007/s10570-019-02299-y | Google Scholar

[B89] Nasrallah D. A. Ibrahim M. A. (2022). Enhancement of Physico-Chemical, Optical, Dielectric and Antimicrobial Properties of Polyvinyl Alcohol/carboxymethyl Cellulose Blend Films by Addition of Silver Doped Hydroxyapatite Nanoparticles. J. Polym. Res. 29, 86. 10.1007/s10965-022-02943-5 10.1007/s10965-022-02943-5 | Google Scholar

[B90] Nicoara A. I. Stoica A. E. Ene D.-I. Vasile B. S. Holban A. M. Neacsu I. A. (2020). *In Situ* and *Ex Situ* Designed Hydroxyapatite: Bacterial Cellulose Materials with Biomedical Applications. Materials 13, 4793. 10.3390/ma13214793 PubMed Abstract | 10.3390/ma13214793 | Google Scholar PMC7663409 33121009

[B91] Núñez D. Cáceres R. Ide W. Varaprasad K. Oyarzún P. (2020). An Ecofriendly Nanocomposite of Bacterial Cellulose and Hydroxyapatite Efficiently Removes Lead from Water. Int. J. Biol. Macromol. 165, 2711–2720. 10.1016/j.ijbiomac.2020.10.055 PubMed Abstract | 10.1016/j.ijbiomac.2020.10.055 | Google Scholar 33069824

[B92] Okuda K. Shigemasa R. Hirota K. Mizutani T. (2022). *In Situ* crystallization of Hydroxyapatite on Carboxymethyl Cellulose as a Biomimetic Approach to Biomass-Derived Composite Materials. ACS Omega 7, 12127–12137. 10.1021/acsomega.2c00423 PubMed Abstract | 10.1021/acsomega.2c00423 | Google Scholar 35449963 PMC9016835

[B93] Oprea M. Voicu S. I. (2020). Recent Advances in Composites Based on Cellulose Derivatives for Biomedical Applications. Carbohydr. Polym. 247, 116683. 10.1016/j.carbpol.2020.116683 PubMed Abstract | 10.1016/j.carbpol.2020.116683 | Google Scholar 32829811

[B94] Palaveniene A. Tamburaci S. Kimna C. Glambaite K. Baniukaitiene O. Tihminlioğlu F. (2019). Osteoconductive 3D Porous Composite Scaffold from Regenerated Cellulose and Cuttlebone-Derived Hydroxyapatite. J. Biomater. Appl. 33, 876–890. 10.1177/0885328218811040 PubMed Abstract | 10.1177/0885328218811040 | Google Scholar 30451067

[B95] Park K. S. Son J. T. Chung H. T. Kim S. J. Lee C. H. Kim H. G. (2003). Synthesis of LiFePO4 by Co-precipitation and Microwave Heating. Electrochem. Commun. 5, 839–842. 10.1016/j.elecom.2003.08.005 10.1016/j.elecom.2003.08.005 | Google Scholar

[B96] Paulraj T. Riazanova A. V. Yao K. Andersson R. L. Müllertz A. Svagan A. J. (2017). Bioinspired Layer-By-Layer Microcapsules Based on Cellulose Nanofibers with Switchable Permeability. Biomacromolecules 18, 1401–1410. 10.1021/acs.biomac.7b00126 PubMed Abstract | 10.1021/acs.biomac.7b00126 | Google Scholar 28323423

[B97] Petrauskaite O. Gomes P. d. S. Fernandes M. H. Juodzbalys G. Stumbras A. Maminskas J. (2013). Biomimetic Mineralization on a Macroporous Cellulose-Based Matrix for Bone Regeneration. BioMed Res. Int. 2013, 1–9. 10.1155/2013/452750 PubMed Abstract | 10.1155/2013/452750 | Google Scholar PMC3791641 24163816

[B98] Pieper C. M. da Rosa W. L. Lund R. G. da Silva A. F. Piva E. Salas M. M. (2020). Biofilms of Cellulose and Hydroxyapatite Composites: Alternative Synthesis Process. J. Bioact. Compatible Polym. 35, 469–478. 10.1177/0883911520951838 10.1177/0883911520951838 | Google Scholar

[B99] Politi Y. Arad T. Klein E. Weiner S. Addadi L. (2004). Sea Urchin Spine Calcite Forms via a Transient Amorphous Calcium Carbonate Phase. Science 306, 1161–1164. 10.1126/science.1102289 PubMed Abstract | 10.1126/science.1102289 | Google Scholar 15539597

[B100] Polshettiwar V. Nadagouda M. N. Varma R. S. (2009). Microwave-assisted Chemistry: A Rapid and Sustainable Route to Synthesis of Organics and Nanomaterials. Aust. J. Chem. 62, 16–26. 10.1071/ch08404 10.1071/ch08404 | Google Scholar

[B101] Qi C. Lin J. Fu L.-H. Huang P. (2018). Calcium-based Biomaterials for Diagnosis, Treatment, and Theranostics. Chem. Soc. Rev. 47, 357–403. 10.1039/c6cs00746e PubMed Abstract | 10.1039/c6cs00746e | Google Scholar 29261194

[B102] Qi C. Musetti S. Fu L.-H. Zhu Y.-J. Huang L. (2019). Biomolecule-assisted Green Synthesis of Nanostructured Calcium Phosphates and Their Biomedical Applications. Chem. Soc. Rev. 48, 2698–2737. 10.1039/c8cs00489g PubMed Abstract | 10.1039/c8cs00489g | Google Scholar 31080987

[B103] Rauch M. W. Dressler M. Scheel H. Van Opdenbosch D. Zollfrank C. (2012). Mineralization of Calcium Carbonates in Cellulose Gel Membranes. Eur. J. Inorg. Chem. 2012, 5192–5198. 10.1002/ejic.201200575 10.1002/ejic.201200575 | Google Scholar

[B104] Rodríguez K. Renneckar S. Gatenholm P. (2011). Biomimetic Calcium Phosphate Crystal Mineralization on Electrospun Cellulose-Based Scaffolds. ACS Appl. Mat. Interfaces 3, 681–689. 10.1021/am100972r 10.1021/am100972r | Google Scholar 21355545

[B105] Saska S. Barud H. S. Gaspar A. M. M. Marchetto R. Ribeiro S. J. L. Messaddeq Y. (2011). Bacterial Cellulose-Hydroxyapatite Nanocomposites for Bone Regeneration. New York, NY: Int. J. Biomater. Article ID 175362. Google Scholar 10.1155/2011/175362 PMC3180784 21961004

[B106] Shi R.-J. Wang T. Lang J.-Q. Zhou N. Ma M.-G. (2022). Multifunctional Cellulose and Cellulose-Based (Nano) Composite Adsorbents. Front. Bioeng. Biotechnol. 10, 891034. 10.3389/fbioe.2022.891034 PubMed Abstract | 10.3389/fbioe.2022.891034 | Google Scholar 35497333 PMC9046606

[B107] Shi S. Chen S. Zhang X. Shen W. Li X. Hu W. (2009). Biomimetic Mineralization Synthesis of Calcium-Deficient Carbonate-Containing Hydroxyapatite in a Three-Dimensional Network of Bacterial Cellulose. J. Chem. Technol. Biotechnol. 84, 285–290. 10.1002/jctb.2037 10.1002/jctb.2037 | Google Scholar

[B108] Sivasankari S. Kalaivizhi R. Gowriboy N. Ganesh M. R. Shazia Anjum M. (2021). Hydroxyapatite Integrated with Cellulose Acetate/polyetherimide Composite Membrane for Biomedical Applications. Polym. Compos. 42, 5512–5526. 10.1002/pc.26242 10.1002/pc.26242 | Google Scholar

[B109] Stoica-Guzun A. Stroescu M. Jinga S. Jipa I. Dobre T. Dobre L. (2012). Ultrasound Influence upon Calcium Carbonate Precipitation on Bacterial Cellulose Membranes. Ultrason. Sonochemistry 19, 909–915. 10.1016/j.ultsonch.2011.12.002 PubMed Abstract | 10.1016/j.ultsonch.2011.12.002 | Google Scholar 22227555

[B110] Stroescu M. Stoica-Guzun A. Jinga S. I. Dobre T. Jipa I. M. Dobre L. M. (2012). Influence of Sodium Dodecyl Sulfate and Cetyl Trimethylammonium Bromide upon Calcium Carbonate Precipitation on Bacterial Cellulose. Korean J. Chem. Eng. 29, 1216–1223. 10.1007/s11814-011-0290-3 10.1007/s11814-011-0290-3 | Google Scholar

[B111] Suchanek W. Yoshimura M. (1998). Processing and Properties of Hydroxyapatite-Based Biomaterials for Use as Hard Tissue Replacement Implants. J. Mat. Res. 13, 94–117. 10.1557/jmr.1998.0015 10.1557/jmr.1998.0015 | Google Scholar

[B112] Tabaght F. E. Azzaoui K. Elidrissi A. Hamed O. Mejdoubi E. Jodeh S. (2021). New Nanostructure Based on Hydroxyapatite Modified Cellulose for Bone Substitute, Synthesis, and Characterization. Int. J. Polym. Mater. Polym. Biomaterials 70, 437–448. 10.1080/00914037.2020.1725758 10.1080/00914037.2020.1725758 | Google Scholar

[B113] Tazi N. Zhang Z. Messaddeq Y. Almeida-Lopes L. Zanardi L. M. Levinson D. (2012). Hydroxyapatite Bioactivated Bacterial Cellulose Promotes Osteoblast Growth and the Formation of Bone Nodules. Amb. Expr. 2, 61. 10.1186/2191-0855-2-61 PubMed Abstract | 10.1186/2191-0855-2-61 | Google Scholar PMC3571908 23174338

[B114] Tommila M. Jokilammi A. Terho P. Wilson T. Penttinen R. Ekholm E. (2009). Hydroxyapatite Coating of Cellulose Sponges Attracts Bone-Marrow-Derived Stem Cells in Rat Subcutaneous Tissue. J. R. Soc. Interface. 6, 873–880. 10.1098/rsif.2009.0020 PubMed Abstract | 10.1098/rsif.2009.0020 | Google Scholar 19324666 PMC2838357

[B115] Tran N. Webster T. J. (2009). Nanotechnology for Bone Materials. WIREs Nanomed Nanobiotechnol 1, 336–351. 10.1002/wnan.23 PubMed Abstract | 10.1002/wnan.23 | Google Scholar 20049801

[B116] Tsuji M. Hashimoto M. Nishizawa Y. Kubokawa M. Tsuji T. (2005). Microwave-assisted Synthesis of Metallic Nanostructures in Solution. Chem. Eur. J. 11, 440–452. 10.1002/chem.200400417 PubMed Abstract | 10.1002/chem.200400417 | Google Scholar 15515072

[B117] Vyroubal R. Saha N. Vesela D. Shah R. Saha P. (2013). Biomimetic Nucleation and Growth of CaCO3 in Bacterial Cellulose Produced by Gluconacetobacter Xylinus (Acetobacter Xylinus). Curr. Opin. Biotechnol. 24, S109. 10.1016/j.copbio.2013.05.336 10.1016/j.copbio.2013.05.336 | Google Scholar

[B118] Wan Y. Hong L. Jia S. Huang Y. Zhu Y. Wang Y. (2006). Synthesis and Characterization of Hydroxyapatite-Bacterial Cellulose Nanocomposites. Compos. Sci. Technol. 66, 1825–1832. 10.1016/j.compscitech.2005.11.027 10.1016/j.compscitech.2005.11.027 | Google Scholar

[B119] Wan Y. Z. Huang Y. Yuan C. D. Raman S. Zhu Y. Jiang H. J. (2007). Biomimetic Synthesis of Hydroxyapatite/bacterial Cellulose Nanocomposites for Biomedical Applications. Mater. Sci. Eng. C 27, 855–864. 10.1016/j.msec.2006.10.002 10.1016/j.msec.2006.10.002 | Google Scholar

[B120] Wang J. Yang C. X. Wan Y. Z. Luo H. L. He F. Dai K. R. (2013). Laser Patterning of Bacterial Cellulose Hydrogel and its Modification with Gelatin and Hydroxyapatite for Bone Tissue Engineering. Soft Mater 11, 173–180. Google Scholar

[B121] Wang X. Tang S. Chai S. Wang P. Qin J. Pei W. (2021). Preparing Printable Bacterial Cellulose Based Gelatin Gel to Promote *In Vivo* Bone Regeneration. Carbohydr. Polym. 270, 118342. 10.1016/j.carbpol.2021.118342 PubMed Abstract | 10.1016/j.carbpol.2021.118342 | Google Scholar 34364595

[B122] Xiao W. Liu J. Chen Q. Wu Y. Dai L. Wu T. (2011). Controllable Mineralization of Calcium Carbonate on Regenerated Cellulose Fibers. Cryst. Res. Technol. 46, 1071–1078. 10.1002/crat.201100261 10.1002/crat.201100261 | Google Scholar

[B123] Yan G. Feng Y. Gao Z. Zeng X. Hong W. Liu W. (2019). Stable and Biocompatible Cellulose-Based CaCO3 Microspheres for Tunable pH-Responsive Drug Delivery. ACS Sustain. Chem. Eng. 7, 19824–19831. 10.1021/acssuschemeng.9b05144 10.1021/acssuschemeng.9b05144 | Google Scholar

[B124] Yang M. Zhen W. Chen H. Shan Z. (2016). Biomimetic Design of Oxidized Bacterial Cellulose-Gelatin-Hydroxyapatite Nanocomposites. J. Bionic Eng. 13, 631–640. 10.1016/S1672-6529(16)60334-7 10.1016/S1672-6529(16)60334-7 | Google Scholar

[B125] Yi L.-J. Li J.-F. Ma M.-G. Zhu Y.-J. (2020). Nanostructured Calcium-Based Biomaterials and Their Application in Drug Delivery. Cmc 27, 5189–5212. 10.2174/0929867326666190222193357 PubMed Abstract | 10.2174/0929867326666190222193357 | Google Scholar 30806303

[B126] Yin N. Chen S.-y. Ouyang Y. Tang L. Yang J.-x. Wang H.-p. (2011). Biomimetic Mineralization Synthesis of Hydroxyapatite Bacterial Cellulose Nanocomposites. Prog. Nat. Sci. Mater. Int. 21, 472–477. 10.1016/s1002-0071(12)60085-9 10.1016/s1002-0071(12)60085-9 | Google Scholar

[B127] Yoshida A. Miyazaki T. Ashizuka M. Ishida E. (2006). Bioactivity and Mechanical Properties of Cellulose/carbonate Hydroxyapatite Composites Prepared *In Situ* through Mechanochemical Reaction. J. Biomater. Appl. 21, 179–194. 10.1177/0885328206059796 PubMed Abstract | 10.1177/0885328206059796 | Google Scholar 16443626

[B128] Yoshida A. Miyazaki T. Ishida E. Ashizuka M. Li P. Zhang K. (2005). Preparation of Cellulose-Carbonate Apatite Composites through Mechanochemical Reaction. Mater 284-286, 855–858. 10.4028/0-87849-961-x.855 10.4028/0-87849-961-x.855 | Google Scholar

[B129] Yuan H. Fernandes H. Habibovic P. de Boer J. Barradas A. M. C. de Ruiter A. (2010). Osteoinductive Ceramics as a Synthetic Alternative to Autologous Bone Grafting. Proc. Natl. Acad. Sci. U.S.A. 107, 13614–13619. 10.1073/pnas.1003600107 PubMed Abstract | 10.1073/pnas.1003600107 | Google Scholar 20643969 PMC2922269

[B130] Yuan Q. Bian J. Ma M.-G. (2021). Advances in Biomedical Application of Nanocellulose-Based Materials: a Review. Cmc 28 (40), 8275–8295. 10.2174/0929867328666201130124501 10.2174/0929867328666201130124501 | Google Scholar 33256574

[B131] Zakharov N. A. Ezhova Z. A. Koval’ E. M. Kalinnikov V. T. Chalykh A. E. (2005). Hydroxyapatite-Carboxymethyl Cellulose Nanocomposite Biomaterial. Inorg. Mat. 41, 509–515. 10.1007/s10789-005-0159-0 10.1007/s10789-005-0159-0 | Google Scholar

[B132] Zhang S. Xiong G. He F. Huang Y. Wang Y. Wan Y. (2009). Characterisation of Hydroxyapatite/Bacterial Cellulose Nanocomposites. Polym. Polym. Compos. 17, 353–358. 10.1177/096739110901700602 10.1177/096739110901700602 | Google Scholar

[B133] Zhu Q. Wang J. Sun J. Wang Q. (2020). Preparation and Characterization of Regenerated Cellulose Biocomposite Film Filled with Calcium Carbonate by *In Situ* Precipitation. BioRes 15, 7893–7905. 10.15376/biores.15.4.7893-7905 10.15376/biores.15.4.7893-7905 | Google Scholar

[B134] Zhu X. D. Fan H. S. Xiao Y. M. Li D. X. Zhang H. J. Luxbacher T. (2009). Effect of Surface Structure on Protein Adsorption to Biphasic Calcium-Phosphate Ceramics *In Vitro* and *In Vivo* . Acta Biomater. 5, 1311–1318. 10.1016/j.actbio.2008.11.024 PubMed Abstract | 10.1016/j.actbio.2008.11.024 | Google Scholar 19121984

[B135] Zhu Y.-J. Chen F. (2014). Microwave-assisted Preparation of Inorganic Nanostructures in Liquid Phase. Chem. Rev. 114, 6462–6555. 10.1021/cr400366s PubMed Abstract | 10.1021/cr400366s | Google Scholar 24897552

[B136] Zimmermann K. A. LeBlanc J. M. Sheets K. T. Fox R. W. Gatenholm P. (2011). Biomimetic Design of a Bacterial Cellulose/hydroxyapatite Nanocomposite for Bone Healing Applications. Mater. Sci. Eng. C 31, 43–49. 10.1016/j.msec.2009.10.007 10.1016/j.msec.2009.10.007 | Google Scholar

